# 抗CD22序贯抗CD19嵌合抗原受体T细胞治疗复发/难治性儿童急性B淋巴细胞白血病2例

**DOI:** 10.3760/cma.j.issn.0253-2727.2022.03.013

**Published:** 2022-03

**Authors:** 剑 张, 小霞 吴, 梦云 郦, 宝全 宋, 莉莉 周, 欣 孔, 惠英 仇, 德沛 吴

**Affiliations:** 苏州大学附属第一医院血液科，国家血液系统疾病临床医学研究中心，江苏省血液研究所，国家卫生健康委员会血栓与止血重点实验室，苏州 215006 National Clinical Research Center for Hematologic Diseases, Jiangsu Institute of Hematology, Hematology Department, The First Affiliated Hospital of Soochow University, Collaborative Innovation Center of Hematology, Suzhou 215006, China

例1，女，9岁，诊断为急性B淋巴细胞白血病（B-ALL），KRAS基因T58I突变。先后接受CCLG-ALL2015、hyper-CVAD（A）方案化疗，均短期复发。FC方案预处理后，行嵌合抗原受体T细胞（CAR-T）治疗。回输鼠源化抗CD19 CAR-T细胞5×10^6^/kg（转染率50.66％）。+2 d患者出现血压下降、反复发热、脉氧下降，胸部CT提示两肺渗出，细胞因子释放综合征（CRS）3级，对症治疗后体温控制，休克纠正。+7 d骨髓评估疾病完全缓解（CR）。

患者于回输后6个月再次出现关节痛，骨髓涂片示原幼细胞占0.615，白血病免疫分型：分析80％幼稚细胞群体，B淋系表达，98.8％ CD19^+^和75.8％ CD22^+^表达。再予IVP、大剂量甲氨蝶呤等方案再诱导化疗。复查骨髓象示原始细胞占0.830，MRD：56％幼稚细胞群体，B淋系表达，98％ CD19^+^和70％ CD22^+^表达。根据免疫分型结果，行CD22 CAR-T序贯人源化CD19 CAR-T细胞治疗。FC方案预处理后，第1、2天（按照40％，60％比例）回输抗CD22 CAR-T细胞，剂量为5×10^6^/kg（感染效率32％），第6、7天（按照40％，60％比例）序贯输注人源化抗CD19 CAR-T细胞，剂量为5×10^6^/kg（感染效率25％）。+7 d复查骨髓象原幼细胞占0.860，MRD 96.8％，期间出现发热、C-反应蛋白（CRP）较基线上升17倍、IL-6较基线上升55倍，评估CRS 2级，予小剂量激素、抗感染等治疗好转。+21 d复查骨髓达CR，原幼细胞占0.015，MRD 0.1×10^−4^。出院后监测血常规、骨髓象及CAR-T拷贝数等指标。患者拒绝造血干细胞移植治疗，3个月后疾病再次复发，疾病进展死亡。

例2，女，11岁。2016年7月外院确诊为B-ALL，融合基因及FISH阴性，染色体49,XX,+X,+8,+21[18]/46,XX[2]，先后接受标危组CCLG-ALL2015、MOLP方案化疗，均获得CR但出现复发。FISH：CRLF2阳性。予Hyper-CVAD（A）再诱导化疗，化疗后复查骨髓原幼细胞占0.040，MRD 1×10^−4^。行CAR-T细胞治疗。FC方案预处理，回输鼠源化抗CD19 CAR-T细胞，回输后患者反复出现发热，CRS 0级，抗感染治疗后好转。回输后评估骨髓：CR，MRD 0.0×10^−4^；CRLF2重排阴性；患者CR_2_后桥接父供女单倍型移植。移植后患者病情稳定，造血重建顺利。移植后9个月患者出现乏力，消瘦明显，复查骨髓象提示原幼细胞占0.790。白血病免疫分型：分析63.2％幼稚细胞群体，CD10、CD19、CD20、CD38、CD22阳性，为B淋系表达，CD19 99.6％，CD22 84.9％。染色体核型：48,X,–X,del（3）（q24q26）,+6,+8,+22[3]/49,idem,+8[7]。FISH：CRLF2阳性。二代测序：TP53 16.1％，CSF3R 53.1％。考虑复发。行供者来源抗CD22 CAR-T序贯人源化抗CD19 CAR-T细胞治疗。回输前原幼细胞占0.060。MRD：分析58％幼稚细胞群体，CD19 99％，CD22 75％。第1、2天回输抗CD22 CAR-T细胞，共计5×10^6^/kg，按照40％、60％回输。第6、7天回输人源化抗CD19 CAR-T细胞，共计5×10^6^/kg，按10％、30％、60％回输。第1、7天予托珠单抗预防CNS。+7 d，患者出现发热、明显胸闷气急，CRP上升21倍、IL-6升高200倍，CRS 2级，予保护脏器﹑水化﹑碱化、利尿等处理后好转。+7 d复查骨髓象示骨髓增生活跃低水平，原幼细胞占0.840，MRD 37.3％。+21 d复查骨髓达CR，原幼细胞占0.010，MRD 0×10^−4^。7个月内监测骨髓始终处于缓解状态，MRD阴性，回输后无GVHD表现。回输后8个月出现全身酸痛﹑发热，血象异常，骨髓穿刺示干抽，后续应用供者淋巴细胞输注、干扰素等治疗，疗效不佳，自动出院后死亡。

